# Synergistic Anti-Tumor Effect of Combining Selective CDK7 and BRD4 Inhibition in Neuroblastoma

**DOI:** 10.3389/fonc.2021.773186

**Published:** 2022-01-27

**Authors:** Yang Gao, Marina Volegova, Nicole Nasholm, Sanjukta Das, Nicholas Kwiatkowski, Brian J. Abraham, Tinghu Zhang, Nathanael S. Gray, Clay Gustafson, Malgorzata Krajewska, Rani E. George

**Affiliations:** ^1^Department of Pediatric Hematology/Oncology, Dana-Farber Cancer Institute and Boston Children’s Hospital, Boston, MA, United States; ^2^Department of Pediatrics, Harvard Medical School, Boston, MA, United States; ^3^Department of Pediatrics, Helen Diller Family Comprehensive Cancer Center, University of California, San Francisco, San Francisco, CA, United States; ^4^Department of Cancer Biology, Dana-Farber Cancer Institute, Boston, MA, United States; ^5^Department of Computational Biology, St. Jude Children’s Research Hospital, Memphis, TN, United States; ^6^Department of Biological Chemistry and Molecular Pharmacology, Harvard Medical School, Boston, MA, United States

**Keywords:** CDK7, BRD4, neuroblastoma, MYCN amplification, resistance

## Abstract

**Purpose:**

Cyclin-dependent kinases (CDKs) that have critical roles in RNA polymerase II (Pol II)-mediated gene transcription are emerging as therapeutic targets in cancer. We have previously shown that THZ1, a covalent inhibitor of CDKs 7/12/13, leads to cytotoxicity in *MYCN*-amplified neuroblastoma through the downregulation of super-enhancer-associated transcriptional upregulation. Here we determined the effects of YKL-5-124, a novel covalent inhibitor with greater selectivity for CDK7 in neuroblastoma cells.

**Experimental Design:**

We tested YKL-5-124 in *MYCN*-amplified and nonamplified neuroblastoma cells individually and in combination with other inhibitors in cell line and animal models. Cell viability, target validation, effects on cell cycle and transcription were analyzed.

**Results:**

CDK7 inhibition with YKL-5-124 did not lead to significant cell death, but resulted in aberrant cell cycle progression especially in *MYCN*-amplified cells. Unlike THZ1, YKL-5-124 had minimal effects on Pol II C-terminal domain phosphorylation, but significantly inhibited that of the CDK1 and CDK2 cell cycle kinases. Combining YKL-5-124 with the BRD4 inhibitor JQ1 resulted in synergistic cytotoxicity. A distinct *MYCN*-gene expression signature associated with resistance to BRD4 inhibition was suppressed with the combination. The synergy between YKL-5-124 and JQ1 translated into significant tumor regression in cell line and patient-derived xenograft mouse models of neuroblastoma.

**Conclusions:**

The combination of CDK7 and BRD4 inhibition provides a therapeutic option for neuroblastoma and suggests that the addition of YKL-5-124 could improve the therapeutic efficacy of JQ1 and delay resistance to BRD4 inhibition.

## Introduction

Neuroblastoma (NB) is an embryonal tumor of the sympathetic nervous system accounting for 15% of all pediatric cancer deaths (1). This cancer of early childhood has a markedly varying clinical course, ranging from spontaneous regression to progression and poor survival of high-risk patients despite aggressive multimodal therapy. About half of high-risk cases display amplification of the *MYCN* oncogene that drives aberrant cell growth and proliferation (2, 3) and results in *MYCN*-driven transcriptional addiction (4, 5). However, no direct approach to target the MYCN protein is currently available and hence indirect approaches to disrupt the regulation of MYCN expression are being developed (6, 7). Inhibition of the bromodomain-containing protein BRD4, which selectively binds to acetylated lysine residues and facilitates the transcriptional activation of target genes, has shown potential in preclinical studies (8, 9) and is currently being tested in clinical trials. However, as with all single-agent targeted therapy, the propensity for resistance is inevitable, underscoring the need for combination treatment strategies at the outset. Moreover, in the remaining 50% of high-risk neuroblastoma patients without tumor *MYCN* amplification, the lack of a unified oncogenic driver makes the design of novel therapies a challenge.

Targeting the aberrant regulation of gene expression through the inhibition of transcription-associated cyclin-dependent kinases (CDKs) is a strategy that is being explored for cancers with transcriptional dependencies, such as NB (4, 10–12). CDK7 regulates both transcription and cell cycle progression (13, 14); as a subunit of the general transcription factor TFIIH which associates with the pre-initiation complex at gene promoters, CDK7 phosphorylates serine 5 and 7 of the carboxy-terminal domain (CTD) of RNA polymerase II (Pol II), facilitating basal transcription (14). Conversely, several studies have reported that CDK7 kinase activity is dispensable for global transcription (15, 16). Through its function as a CDK-activating kinase (CAK), CDK7 also associates with Cyclin H and Mat1 to induce T-loop phosphorylation of not only other transcription-associated CDKs such as CDK9 (10) but also cell cycle CDKs such as CDKs 1 and 2, thereby controlling cell cycle progression.

We previously reported that inhibition of CDK7 with THZ1, a covalent inhibitor of CDK7/12/13 (17), leads to significant tumor regression in *MYCN*-amplified NB (4). The cytotoxic effect of THZ1 was associated with inhibition of *MYCN*-dependent transcriptional amplification and preferential targeting of super-enhancer (SE)-associated genes. However, inhibition of CDK12/13 function with THZ1 also likely contributed to its therapeutic effect. Given the multiple targets of THZ1, we asked whether selective inhibition of CDK7 would be sufficient to induce cytotoxicity in NB and whether its therapeutic effects could be attributed to inhibition of CDK7-mediated transcription, cell cycle progression or both. We therefore took advantage of the covalent inhibitor YKL-5-124, with a wide window of selectivity for CDK7 over CDK12 and CDK13 (18). We demonstrate that inhibition of CDK7 with YKL-5-124 primarily affects cell cycle progression and results in proliferative arrest which was especially prominent in *MYCN* amplified NB cells. Through combinatorial screening, we identify that YKL-5-124 is synergistic with inhibitors of BRD4, leading to a profound decrease in Pol II transcription and G2-M cell cycle arrest. Finally, we demonstrate that the synergistic cytotoxicity observed *in vitro* translates into anti-tumor effects *in vivo*, supporting the therapeutic potential of targeting CDK7 and BRD4 in combination in NB.

## Materials and Methods

### Cell Culture

Human neuroblastoma (NB) cells (Kelly, IMR-32, IMR-5, LAN-5, SK-N-DZ, SK-N-BE2, SH-SY5Y, CHLA-20, and SK-N-AS) were obtained from the Children’s Oncology Group cell line bank and genotyped at the DFCI Core Facility. NB cells were grown in RPMI (Invitrogen) supplemented with 10% FBS and 100 U/ml penicillin/streptomycin (Invitrogen). Human lung (IMR-90) and skin fibroblasts (BJ) were kindly provided by Dr. Richard Gregory (Boston Children’s Hospital). IMR-90 and BJ cells were grown in DMEM (Invitrogen) supplemented with 10% FBS and 100 U/ml penicillin/streptomycin. All cell lines were routinely tested for mycoplasma.

### Compounds

YKL-5-124 (18), THZ531 (19) and THZ1 (4) were prepared by Dr. Nathanael Gray’s laboratory. JQ1 (20) was obtained from J. Qi, DFCI. RG-108, C646, UNC0638, GSK-J4, GSK343, SAHA, Iniparib, iBET726, iBET151, LDC000067, ZXH-03-026 were purchased from SelleckChem.

### Cell Viability and Drug Combination Assays

Cells were plated in 96-well plates at a seeding density of 4 x 10^3^ cells/well. After 24 h, cells were treated with increasing concentrations of YKL-5-124, THZ1, JQ1, iBET726, iBET151 or ZXH-03-026 (10 nM to 10 μM). DMSO without compound served as a negative control. After 72 h incubation, viability was analyzed using the CellTiter-Glo Luminescent Cell Viability Assay (Promega), according to the manufacturer’s instructions. All proliferation assays were performed in biological triplicates. Drug concentrations that inhibited 50% of cell growth (IC_50_) were determined using a nonlinear regression curve fit using GraphPad Prism 6 software. For the synergy analyses compounds were added simultaneously at the constant ratio of 1: 4, starting at 100 nM and 400 nM for YKL-5-124 and JQ1 or YKL-5-124 and other compounds listed above. Synergy was assessed using the Chou-Talalay combination index (CI) method. CI method determines whether or not a two-drug combination demonstrates synergy, where the combined effect is greater than the sum of the individual effects (21, 22). Specifically, a CI < 1 indicates synergy, a CI > 1, antagonism, and a CI = 1 additivity. Synergy was defined as “strong” when CI values were < 0.3 as defined in the literature (22). The CI scores were computed for the range of concentrations of drug combinations and fraction affected (Fa) versus combination index (Fa-CI) plots were generated to determine the extent of synergy if any, using CompuSyn software (https://www.combosyn.com/).

### Western Blotting

Cells were collected by trypsinization and lysed at 4°C in NP40 buffer (Invitrogen) supplemented with complete protease inhibitor cocktail (Roche), PhosSTOP phosphatase inhibitor cocktail (Roche) and PMSF (1 mM). Protein concentrations were determined with the Biorad DC protein assay kit (Bio-Rad). Whole-cell protein lysates were resolved on 4%–12% Bis-Tris gels (Invitrogen) and transferred to nitrocellulose membranes (Bio-Rad). After blocking nonspecific binding sites for 1 h using 5% dry milk (Sigma) in Tris-buffered saline (TBS) supplemented with 0.2% Tween-20 (TBS-T), membranes were incubated overnight with primary antibody at 4°C. Chemiluminescent detection was performed with the appropriate secondary antibodies and developed using Genemate Blue ultra autoradiography film (VWR).

### Fluorescence-Activated Cell Sorting Analysis (FACS)

For cell cycle analysis, cells were treated with DMSO or YKL-5-124 (100 nM) or JQ1 (400 nM) or a combination of both. After 8, 24, 48 and 72 h, cells were trypsinized and fixed in ice-cold 70% ethanol overnight at -20°C. After washing with ice-cold phosphate-buffered saline (PBS), cells were treated with 100 µg/mL RNase A (Sigma-Aldrich) in combination with 50 µg/ml propidium iodide (PI, BD Biosciences). For EdU analysis, cells were treated with DMSO, YKL-5-124 (100 nM), JQ1 (400 nM), or combinations of both for 24 h. Cells were pulsed with 10 µM of 5-ethynyl-2’-deoxyuridine (EdU) for 2 h and subsequently collected by trypsinization. Cells were fixed and stained for EdU incorporation using Click-iT EdU Alexa Fluor 647 Flow Cytometry Assay Kit (Thermo Fisher) according to the manufacturer’s instructions. After EdU staining, cells were resuspended in Click-iT saponin-based permeabilization and wash reagent (Thermo Fisher) with 50 µg/mL propidium iodide (PI, BD Biosciences) and 100 µg/mL RNase A (Sigma-Aldrich). All samples were analyzed on an LSR Fortessa (Becton Dickinson) using FACSDiva software (Becton Dickinson). A minimum of 50,000 events was counted per sample and used for further analysis. Data were analyzed using FlowJo software.

### Target Engagement Assay

Tumor tissue or NB cells treated with THZ1, YKL-5-124, or DMSO for 6 h at the indicated doses were lysed in NP40 buffer (Invitrogen) containing complete protease inhibitor cocktail (Roche), PhosSTOP phosphatase inhibitor cocktail (Roche) and PMSF (1 mM). To IP CDK7, CDK12 or cyclin H, 1 mg of total protein was incubated with 1 µM of biotin-THZ1 at 4°C overnight. Subsequently, lysates were incubated with streptavidin agarose (30 µl) for 2 h at 4°C. Agarose beads were washed 3x with cell lysis buffer and boiled for 10 min in 2x gel loading buffer. Proteins were resolved by western blotting. 50 µg of total protein was used as a loading control.

### Antibodies

The following antibodies were used: Pol II CTD S2 (Bethyl cat# A300-654A, 1:10,000); Pol II CTD S5 (Bethyl cat# A300-655A, 1:10,000); Pol II (Santa Cruz cat# sc-899, 1:1000); cleaved PARP (Cell Signaling cat# 9541, 1:1000); Cleaved Caspase-3 (Cell Signaling cat# 9661, 1:1000); GAPDH (Cell Signaling cat# 2118S, 1:4000); CDK12 (Cell Signaling cat# 11973S, 1:1000); CDK7 (Santa Cruz cat# sc-365075, 1: 1000); MYCN (Cell Signaling cat# 9405S, 1: 1000); CDK1 (Santa Cruz cat# sc-53219, 1:1000), pCDK1 (Cell Signaling cat# 9114, 1:1000); CDK2 (Cell Signaling cat#2546, 1:1000), pCDK2 (Cell Signaling cat# 2561, 1:1000); cyclin H (Santa Cruz #sc-609, m1:1000).

### RT-PCR

Total RNA was isolated with the RNAeasy Mini kit (QIAGEN). One µg of purified RNA was reverse transcribed using SuperScript IV VILO master mix (Invitrogen) following the manufacturer’s protocol. Quantitative PCR was carried out using the QuantiFast SYBR Green PCR kit (Qiagen) and analyzed on an Applied Biosystems StepOne Real-Time PCR System (Life Technologies). Each individual biological sample was qPCR-amplified in technical triplicate and normalized to GAPDH as an internal control. Relative quantification was calculated according to the ΔΔCt relative quantification method. Error bars indicate ± SD of three replicates. Primer sequences are available on request.

### RNA-Sequencing

Cells were treated with DMSO or YKL-5-124 (100 nM) or JQ1 (400 nM) or combination of both for 4 h. RNA extraction was performed with mirVana miRNA isolation kit (Invitrogen) following the manufacturers’ instructions. Total RNA was treated with DNase I (Invitrogen). Sequencing libraries were prepared with the Illumina TruSeq stranded mRNA kit (Illumina) following the manufacturers’ instructions and with spike-in controls for sample normalization. All samples were sequenced on an Illumina SE75 sequencer.

### RNA-Sequencing Data Processing

RNA-seq reads were aligned to a reference genome comprised of the non-random chromosomes of the hg19 human reference plus the ERCC92 probe sequences using tophat v2.1.1 (23) with –no-novel-juncs and -G set to a database of RefSeq genes downloaded 7/5/2017 that also contain the ERCC probe coordinates as separate chromosomes. Per-gene expression was quantified in these same RefSeq genes and ERCC probes using htseq-count (24) using -m intersection-strict.

### Differential Expression Analysis

Differential gene expression analysis was performed using DESeq2 in R. To detect differentially expressed genes (DEGs) in each sample, raw read counts from the RNA-seq data were imported to DESeq2. Spike-in read counts were used for each sample, and the size factors were calculated to normalize the library sizes using the estimateSizeFactors (DESeq2) function available in R. A transcript with an absolute log2 fold-change ≥ 1.5 and an adjusted *P*-value ≤ 0.01 was considered significant.

### Analysis of Gene Expression Changes in NB Cells With Drug Treatment

Changes in gene expression in NB cells treated with single agents (YKL-5-124 or JQ1) or the combination were compared. The genes that showed changes in gene expression with a FC ≥ 0.5 (FDR ≤ 0.01) with either single agent compared with DMSO control and a FC ≤ -1.0 with the combination were selected for further analysis.

### Enrichment Analysis

Gene ontology enrichment for selected gene sets was performed by the Enrichr program (https://amp.pharm.mssm.edu/Enrichr/) and GSEA v2.1.0 software. All GO terms were ranked based on the Enrichr combined score, calculated by multiplying the adjusted *P*-value with the z-score using the Fisher’s exact test. The Fisher’s exact test was used to determine significant overlaps between the queried gene sets and other publicly available datasets. Enrichment of gene sets was considered significant for an adjusted *P*-value ≤ 0.01. GSEA sets with an FDR ≤ 0.3 and a *P* value ≤ 0.05 were considered significant.

### Animal Studies

All mouse experiments were performed using subcutaneous injections of 1x10^6^ *MYCN*-amplified cells (IMR-32) or *MYCN*-amplified patient-derived xenograft (SFNB17) into 4-6-week-old recipient NOD.Cg-*Prkdc^scid^Il2rg^tm1Wjl^*/SzJ (NSG; The Jackson Laboratory 005557) female mice. When a tumor volume of 200 mm^3^ was reached, mice were randomly assigned into treatment groups, with the volume being equal between groups. For the efficacy arm, mice were treated with 2.5 mg/kg YKL-5-124 (diluted in D5W + 10% DMSO), 25 mg/kg JQ1 (diluted in 10% HP-beta-cyclodextrin) or vehicle control (5DW +10% DMSO + 10% HP-beta-cyclodextrin). For the IMR-32 cell xenograft experiments, mice were dosed daily by intraperitoneal (IP) injection for 24 days, however because of significant toxicity in this experiment, the dosing for the SFNB17 PDX experiments was three times per week for 25 days (10 doses) by IP injection. Tumor size and body weight were monitored three times per week and tumor volume was calculated using the ellipsoid formula (*1/2(max diameter x min diameter^2^*). For the IMR-32 cell experiment, mice that reached protocol limits for body conditioning score <2 (25) were removed from the survival analysis, but remained in the tumor size analysis (**Figures 5A, B**). Once tumors reached 2 cm in diameter, the mice were euthanized according to approved animal protocols. For the pharmacodynamic arm, mice were treated for 7 days and harvested 1 h after the final treatment. For both analyses, tumors were either fixed in 10% neutral buffered formalin, or snap-frozen and stored at -80°C until further analysis. All animal experiments were conducted according to approved protocols by the Institutional Animal Care Use Committee (IACUC) of the University of California, San Francisco (UCSF).

### Immunohistochemistry

Formalin-fixed tumors were paraffin-embedded and sliced into 5-micron slices by the UCSF Brain Research and Tumor Core. After citrate-based antigen retrieval (Vector Laboratories), tumor sections were stained with H&E, Cleaved Caspase 3 (CST 9661; 1:200), Ki67 (Invitrogen MA5-14520;1:200), or MYCN (CST 51705; 1:600). Development with DAB (Vector Laboratories) was performed according to manufacturer’s instructions. Cleaved Caspase 3, Ki67 and MYCN slides were counterstained with Gill’s II Hematoxylin. Slides were imaged at 40X magnification using a Leica DMi8 inverted microscope.

## Results

### YKL-5-124 Suppresses CDK7 CAK Activity and Leads to G1 Arrest in NB Cells

**Figure 1:**
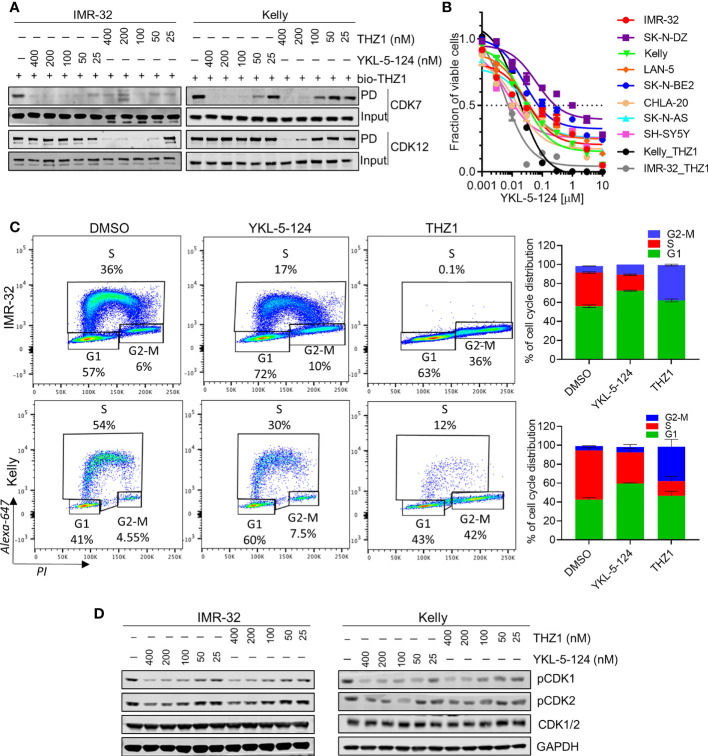
Selective CDK7 inhibition with YKL-5-124 leads to aberrant cell cycle progression in NB cells. **(A)** Analysis of target engagement in NB cells following THZ1 or YKL-5-124 treatment. Cells were treated with THZ1, YKL-5-124 or DMSO for 6 h at the indicated doses and cell lysates were incubated with 1 μM of biotinylated THZ1 (bio-THZ1) overnight. Western blot (WB) analysis of CDK7 and CDK12 in the immunoprecipitate (PD). **(B)** Dose-response curves for NB cells treated with increasing concentrations of YKL-5-124 or THZ1 for 72 h. Cytotoxicity is reported as percent cell viability relative to DMSO-treated cells. Data represent mean ± SD; *n* = 3. **(C)** Flow cytometry analysis of EdU staining in NB cells treated with 100 nM THZ1, 100 nM YKL-5-124 or DMSO for 24 h (left). The percentages of living cells in each phase of the cell cycle are shown. Quantification of staining (right), data represents mean ± SD; *n* = 3. **(D)** WB analysis of pCDK1 (Thr161), pCDK2 (Thr160) and total CDK1/2 in NB cells treated with YKL-5-124 or THZ1 at indicated doses for 6 h.

To compare the on-target activity of the more selective CDK7 inhibitor, YKL-5-124 (18) with THZ1 in *MYCN*-amplified NB cells, we first evaluated the extent of target engagement using a biotinylated derivative of THZ1 (bio-THZ1) (**Figure 1A**). In contrast to THZ1, YKL-5-124 treatment strongly inhibited the pulldown of CDK7 with bio-THZ1 while completely sparing CDK12 even at relatively high doses (400 nM) and confirming its on-target selectivity for CDK7 over CDK12. YKL-5-124R, a cysteine non-reactive analog of YKL-5-124 that does not covalently target the protein (18), exhibited at least 40 times lower anti-proliferative activity (**Supplementary Figure 1A**), indicating that covalent bond formation is a significant contributor to the cellular potency of YKL-5-124. Next, we assessed the effect of YKL-5-124 on cell viability in a panel of NB cell lines. Unlike THZ1, which was highly cytotoxic at low nanomolar concentrations (IC_50_, 6-9 nM) in *MYCN*-amplified NB cells (4), YKL-5-124 appeared to be cytostatic (IC_50_, 8-60 nM), causing growth arrest rather than cell death in both *MYCN*-amplified and nonamplified NB cells (**Figure 1B**). To address this difference, we compared the effect of YKL-5-124 and THZ1 on cell cycle progression using pulse-labeling with 5-ethynyl-20-deoxyuridine (EdU) combined with propidium iodide staining. YKL-5-124 treatment led to predominant accumulation of cells in the G1 phase of the cell cycle, with a concomitant decrease in the proportion of cells in S-phase in *MYCN*-amplified cells, whereas in nonamplified cells these effects were less prominent (**Figure 1C** and **Supplementary Figures 1B, C**). Conversely, the loss of S-phase cells in THZ1-treated cells was accompanied by G2-M arrest. These perturbations in cell cycle progression following YKL-5-124 treatment led us to test whether CDK7-mediated phosphorylation of cell cycle-associated CDKs could be affected. Indeed, we observed a dose- and time-dependent decrease in CDK1 and 2 phosphorylation in YKL-5-124-treated cells, (**Figure 1D**). Thus, the CDK7 inhibitor YKL-5-124 displayed phenotypic effects that were distinct from those of the CDK7/12/13 inhibitor THZ1 in NB cells. Consistent with previous reports in other models (18, 26), YKL-5-124 primarily induced G1 proliferation arrest accompanied by impaired DNA synthesis, likely through inhibition of the CAK activity of CDK7.

### Selective CDK7 Inhibition With YKL-5-124 Is Not Sufficient to Cause Downregulation of RNA Pol II Transcription in NB Cells

**Figure 2:**
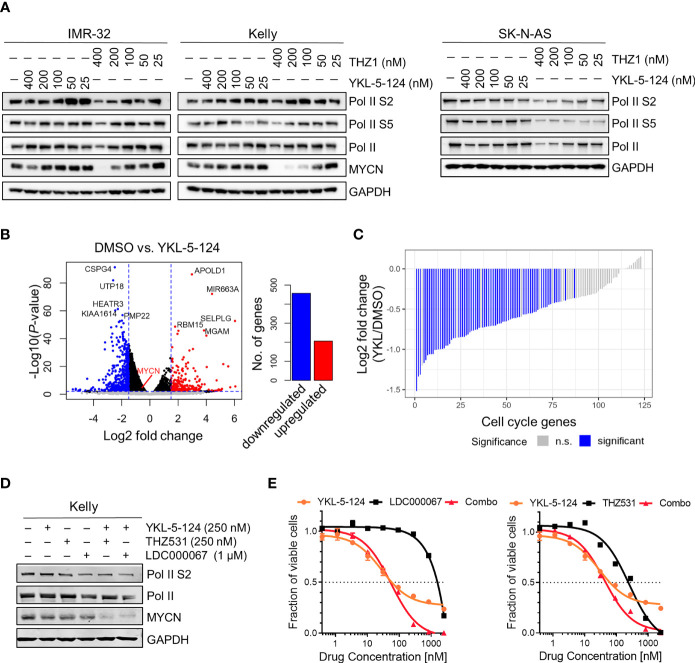
YKL-5-124 does not induce global transcription downregulation in NB cells. **(A)** WB analysis of RNA Pol II phosphorylation and MYCN levels in NB cells following treatment with THZ1 and YKL-5-124 at the indicated doses for 6 h. **(B)** Volcano plot representation of differentially expressed genes following treatment with YKL-5-124 100 nM for 4 h (*left*). Fold change is represented in log2 scale (x-axis) and the −log10 *P*-value depicted on the y-axis (FDR < 0.01 and log2 FC >1.5). Bar plot depicting the numbers of up- and downregulated genes (*right*). **(C)** Waterfall plot depicting the log2 fold-change in gene expression of cell cycle genes in IMR-32 cells treated with YKL-5-124, as in **(B)**. **(D)** WB analysis of the indicated proteins in Kelly NB cells treated with YKL-5-124 in combination with LDC000067 or THZ531 at the indicated concentrations for 6 h. **(E)** Dose-response curves for Kelly cells treated with increasing concentrations of YKL-5-124 in combination with LDC000067, left (1:4 ratio) or THZ531, right (1:1 ratio) for 72 h. Cytotoxicity is reported as percent cell viability relative to DMSO-treated cells. Data represent mean ± SD; *n* = 3.

CDK7 inhibition with THZ1 has been shown to reduce the expression of SE-associated genes and induce global transcriptional shutdown in several cancer models, including NB (4, 11, 12). Unlike THZ1, we observed that YKL-5-124 treatment had only a minor effect on Pol II phosphorylation in NB cells, indicating that global transcription was not affected (**Figure 2A**). To identify the genes whose expression was affected by YKL-5-124 treatment, we performed RNA-sequencing in NB cells exposed to 100 nM YKL-5-124 for 4 hours. In stark contrast to our previous study with THZ1 (4), YKL-5-124 treatment in NB cells did not lead to global transcriptional shutdown, but rather, led to minor changes in gene expression, with 206 and 457 up- and downregulated genes, respectively (log2 fold change >1.5, FDR ≤ 0.01) (**Figure 2B** and **Supplementary Table 1**). Consistent with previous reports (18, 26), we also observed decreased expression of cell cycle-associated genes (GSEA: KEGG_CELL_CYCLE) in these cells (**Figure 2C** and **Supplementary Table 2**). Compared with THZ1, YKL-5-124 failed to completely reduce the expression of SE-associated genes, including *MYCN* (**Figures 2A, B**). The discrepancy between results obtained with YKL-5-124 and THZ1 suggested that concomitant inhibition of other transcriptional CDKs might be required to inhibit global Pol II transcription. Combinations of YKL-5-124 and LDC000067 (27), a CDK9 inhibitor or THZ531 (19) a CDK12/13 inhibitor, led to reduced Pol II phosphorylation and had an additive impact on cell viability (**Figures 2D, E**). In addition, these combinations also resulted in more pronounced decrease in MYCN expression (**Figure 2D**). Together, these results demonstrate that selective CDK7 inhibition is not sufficient to inhibit global Pol II transcription.

### Combined CDK7 and BRD4 Inhibition Synergistically Suppresses Proliferation of NB Cells

**Figure 3:**
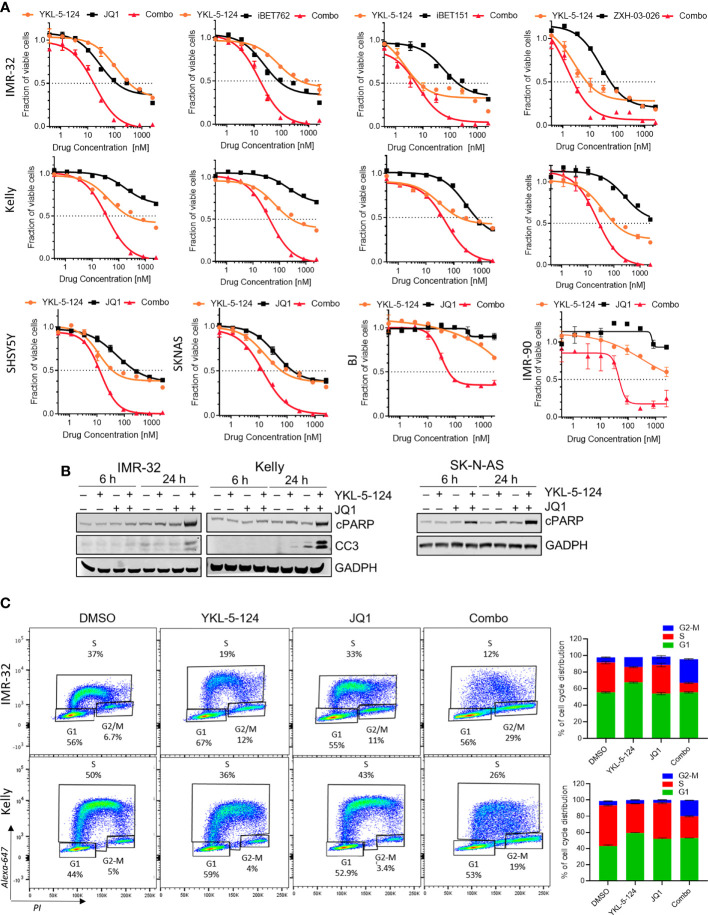
The addition of BRD4 inhibitors to YKL-5-124 leads to cytotoxicity in NB cells. **(A)** Dose-response curves for NB cells and BJ and IMR-90 fibroblast cells treated with YKL-5-124 and the indicated BRD4 inhibitors (JQ1, iBET762, iBET151) or degrader (ZXH-03-026), singly or in combination for 72 h. For the combination (combo), cells were treated with YKL-5-124 and BRD4 inhibitor (as indicated on the graph) at a constant ratio of 1:4. **(B)** WB analysis of apoptosis markers in NB cells treated with 100 nM YKL-5-124, 400 nM JQ1 or the combination at the indicated time points. **(C)** Flow cytometry analysis of EdU staining in NB cells treated with DMSO, 100 nM YKL-5-124, 400 nM JQ1 or the combination for 24 h (left). Percentages of living cells in each phase of the cell cycle are shown. Quantification of staining (right), data represent mean ± SD; *n* = 3.

As a single agent, YKL-5-124 was not sufficient to cause substantial cytotoxicity in NB cells. We therefore asked whether combining YKL-5-124 with a second agent could enhance cytotoxicity. To identify such combinations, we screened a small molecule library of inhibitors of epigenetic targets, including enzymes involved in chromatin methylation and histone acetylation/deacetylation, as well as epigenetic “readers” (**Supplementary Figure 2**). Synergistic cytotoxicity as calculated by the Chou-Talalay Combination Index (CI) (22), was observed when YKL-5-124 was combined with inhibitors targeting Bromo- and Extra-Terminal domain proteins (BETis) in *MYCN*-amplified and nonamplified NB cells (**Figure 3A**, **Supplementary Figures 2**, **3A** and **Supplementary Table 3**). Several inhibitors with unique chemical scaffolds (JQ1, iBET762 and iBET151) (20, 28, 29) exhibited similar levels of synergy, thus substantiating a direct role for BRD(s) in mediating these effects. In addition, a recently reported BRD4-selective chemical degrader, ZXH-3-026, recapitulated the potent synergistic cytotoxicity seen with the combination (**Figure 3A**, **Supplementary Figure 3A** and **Supplementary Table 3**), confirming that on-target inhibition of BRD4 underlies the synergistic interaction with YKL-5-124. In human lung (IMR-90) and skin (BJ) fibroblast cells, the combination treatment was less cytotoxic than that observed in NB cells (**Figure 3A**). The lowest cytotoxic doses of the combinations of YKL-5-124 and JQ1 or ZXH-3-026 (100 nM and 400 nM, respectively) were selected to further characterize the synergistic cellular effects. The combination of YKL-5-124 and JQ1 led to apoptotic cell death, manifested by an increase in cleaved PARP and cleaved caspase 3 levels (**Figure 3B**), which was also seen with ZXH-3-026 (**Supplementary Figure 3B**). The addition of JQ1 potentiated the effect of YKL-5-154 on DNA replication and caused G2-M cell cycle arrest (**Figure 3C**). In summary, the combination of CDK7 and BRD4 inhibition synergistically reduced NB cell proliferation, leading to G2-M arrest and apoptotic cell death.

### The Combination of YKL-5-124 and JQ1 Suppresses RNA Pol II-Mediated Transcription

**Figure 4:**
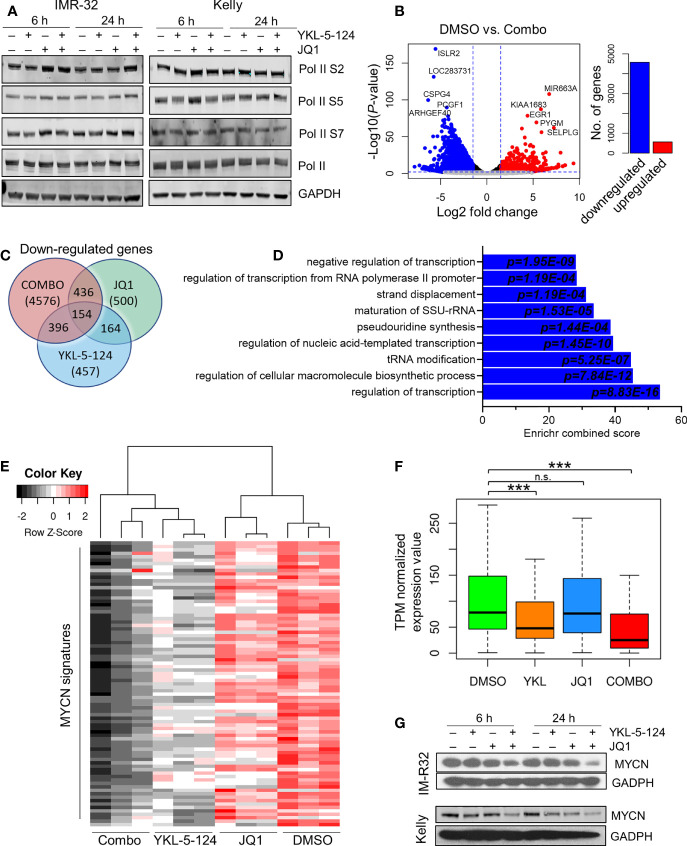
Combined inhibition of CDK7 and BRD4 affects RNA Pol II transcription. **(A)** WB analysis of Pol II phosphorylation in NB cells treated with 100nM YKL-5-124, 400 nM JQ1, or the combination at the indicated concentrations for the indicated times. **(B)** Volcano plot of differentially expressed genes following treatment with the combination of 100 nM YKL-5-124 and 400 nM JQ1 for 4 h (*left*). The fold changes are represented in log2 scale (x-axis), and the −log10 *P*-value depicted on the y-axis (FDR < 0.01 and log2 FC >1.5). Bar plot depicts the numbers of up- and downregulated genes (*right*). **(C)** Venn diagram representing downregulated genes (FC >1.5 and FDR ≤ 0.01) in NB cells treated with 100 nM YKL-5-124, 400 nM JQ1 or the combination. **(D)** GO enrichment analysis of genes significantly downregulated in NB cells after treatment as in **(B)**. **(E)** Heat map of expression values of the MYCN gene signature in RNA-seq analysis of NB cells treated with 100 nM YKL-5-124, 400 nM JQ1, or the combination for 4 h. The MYCN 87-gene signature was established in Valentijn et al. (30). **(F)** Box plots comparing gene expression changes in the MYCN signature within the indicated data sets. The center line indicates the median for each data set. Significance was determined by Welch’s two-sample t-test (***P < 0.001); n.s., not significant. **(G)** WB analyses of MYCN expression in NB cells treated with 100 nM YKL-5-124, 400 nM JQ1 or the combination at the indicated concentrations for the indicated times.

We next analyzed the downstream consequences of the combination of YKL-5-124 and JQ1 in NB cells. Treatment at the lowest synergistic doses (100 nM YKL-5-124 and 400 nM JQ1) had no effect on Pol II CTD phosphorylation (**Figure 4A**). To identify transcriptional changes induced by dual CDK7 and BRD4 inhibition, we performed RNA-sequencing in NB cells treated with the combination for 4 hours. The relatively short treatment time was chosen to identify the immediate transcriptional changes which could explain the synergistic effect without confounding cytotoxicity issues. Similar to results with YKL-5-124 (**Figure 2B**), JQ1 single-agent treatment had modest effects on gene expression with 383 and 500 up- and downregulated transcripts respectively (log2 fold change >1.5, FDR ≤ 0.01) (**Supplementary Figures 4A, B**). However, the combination exhibited a potent effect on gene expression and led to significant downregulation of 4576 genes, whereas 564 were upregulated (log2 fold change >1.5, FDR ≤ 0.01) (**Figures 4B, C** and **Supplementary Table 1**). Gene ontology (GO) enrichment analysis and GSEA of the downregulated genes revealed gene sets associated with transcription, ribosome biogenesis, and tRNA processing (**Figure 4D** and **Supplementary Tables 1**, **2**). Because we did not observe repression of *MYCN*-driven global transcriptional amplification following YKL-5-124 treatment, we next asked whether this compound singly or in combination had any effect on a previously published functional *MYCN* gene signature that was highly correlated with MYCN expression in a cohort of 88 primary tumors (30). Of the 157 genes that made up this signature, 87 genes were positively correlated with MYCN expression. YKL-5-124 led to downregulation of the positively correlated *MYCN* signature (log2 fold change >1, FDR ≤ 0.01; n=17/87), whereas no such effect was seen with JQ1. However, the combination of the two agents led to a much more pronounced downregulation of the *MYCN* gene signature (log2 fold change >1, FDR ≤ 0.01; n=60/87) (**Figures 4E, F**). In line with this result, MYCN expression levels were also significantly decreased in cells treated with combination (**Figure 4G**).

To further elucidate the mechanisms underlying the strong synergy observed with the combination, we hypothesized that combined CDK7 and BRD4 inhibition might block the emergence of pro-survival signaling that followed individual inhibition of each target. Therefore, we asked whether a gene signature that was upregulated with each single agent was subsequently downregulated by the combined CDK7 and BRD4 inhibition. We identified 14 genes that were upregulated with YKL-5-124 (FDR < 0.1; log2 FC >1) and downregulated with the combination (**Supplementary Figure 4C** and **Supplementary Table 1**); however, none of these genes were associated with pro-survival signaling and were excluded from further inquiry. Of the 383 transcripts that were upregulated following single agent JQ1 treatment, a distinct gene signature comprising 344 genes was identified, which was subsequently downregulated with the combination (**Supplementary Figures 4C, D** and **Supplementary Table 1**). GO analysis revealed that these genes were primarily involved in G2-M transition and chromosome segregation (**Supplementary Figures 4E–G**). These data suggest that cells treated with JQ1 as a single agent become especially susceptible to CDK7 inhibition, owing to the upregulation of critical growth-promoting genes and which are in turn suppressed by YKL-5-124, thus contributing to the improved therapeutic efficacy.

### The Combination of YKL-5-124 and JQ1 Suppresses the Growth of NB in Subcutaneous and Patient-Derived Xenograft Models

**Figure 5:**
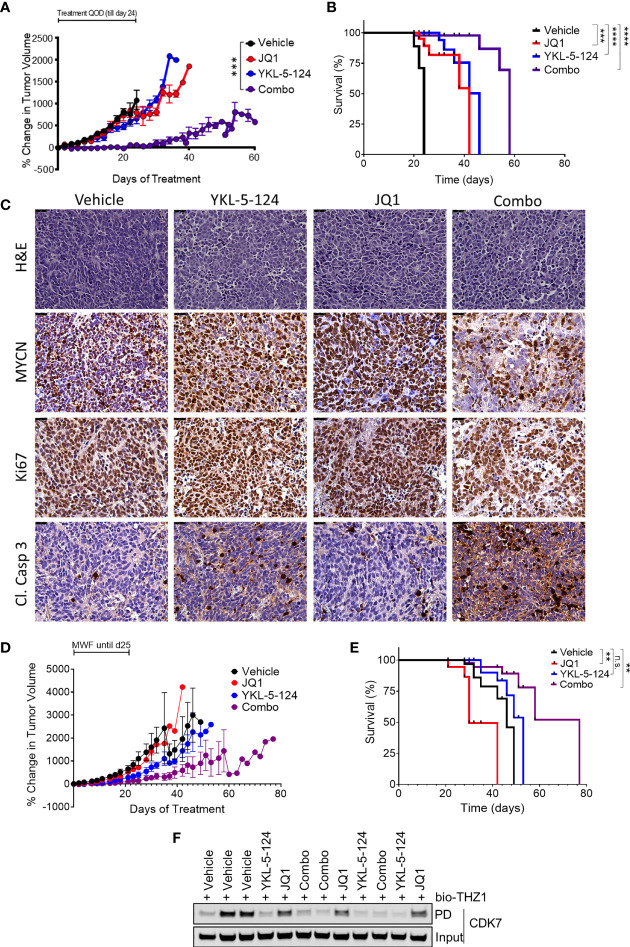
The combination of YKL-5-124 and JQ1 leads to improved therapeutic efficacy *in vivo*. **(A)** Tumor volumes of IMR-32 NB xenografts in NSG mice treated with 2.5 mg/kg YKL-5-124, 25 mg/kg JQ1, JQ1 and YKL-5-124 or vehicle control (5DW +10% DMSO + 10% HP-beta-cyclodextrin) QOD including weekends for 24 days. Mean ± SEM values are presented. (n=5, all arms) ***P < 0.001, as compared to vehicle. **(B)** Kaplan-Meier survival curves for the experiment described in **(A)** but with mice with BCS<2 excluded. ***P < 0.001, ****P < 0.0001 (log-rank test). **(C)** Immunohistochemical (IHC) analysis of morphology (hematoxylin & eosin, H&E), proliferation (Ki67), apoptosis (cleaved caspase 3) and MYCN in tumors harvested from animals treated as described in **(A)** for 7 days. Magnification, 40X. **(D)** Tumor volumes of PDX tumors in NSG mice treated with the indicated agents at similar doses as in **(A)** or vehicle control three times per week for 28 days (14 total treatments). Mean ± SEM values are presented. (vehicle n=8, other arms n=6). **(E)** Kaplan-Meier survival curves for the experiment described in **(C)** **P < 0.01, ns, not significant (log-rank test). **(F)** Analysis of target engagement in tumors from mice treated as described in **(C)** Lysates prepared from homogenized tumor tissue were incubated with bio-THZ1 for 24 h at 4°C and immunoprecipitated proteins analyzed for CDK7 by WB.

The synergistic effects of combining YKL-5-124 with JQ1 *in vitro* prompted us to assess the efficacy of this combination in *in vivo* models of NB. To determine optimal drug dosing, we first conducted maximum tolerated dose (MTD) and pharmacodynamics (PD) studies of YKL-5-124 and JQ1 combinations in non-tumor-bearing NSG mice. Daily dosing of YKL-5-124 at 2.5 mg/kg and JQ1 at 25 mg/kg was tolerable in the short-term, but prolonged administration led to toxicity. However, the combination at the same doses administered on alternate days (QOD) led to no significant weight loss (**Supplementary Figure 5A**). Target engagement assays were performed using spleen and liver tissue at 12 h after the seventh dose. Biotin-THZ1 was able to immunoprecipitate CDK7 (cyclin H as a surrogate) from both tissues from vehicle-treated mice, whereas the combination led to a near complete blockade of such pulldown, indicating substantial CDK7 engagement using this dosing regimen (**Supplementary Figure 5B**). We next carried out *in vivo* efficacy studies in an established human NB cell line (IMR-32) subcutaneous xenograft model. Tumor-bearing mice were treated with vehicle or the combination at the MTD dose and frequency for 24 days. We observed that combination therapy in tumor-bearing mice was more toxic than in tumor-naïve control animals. Mice experiencing excessive, early weight loss were excluded from the survival analysis, but tumor size was still evaluated (**Figure 5A**). Mice that did not experience 15% weight loss were monitored until they reached the endpoint. While JQ1 treatment led to no significant delay in tumor growth and YKL-5-124 alone to a moderate delay, the combination led to a significant suppression of tumor growth, although regrowth was seen upon treatment termination. In animals that were not excluded due to toxicity, both YKL-5-124 and JQ1 single-agent treatment significantly extended survival compared to vehicle controls (p<0.0001); the combination further prolonged tumor-free survival compared to single-agents (p=0.0005) (**Figure 5B**). Of note, although the mice treated with the combination exhibited weight loss, they regained weight when treatments were completed. Tumor biopsy specimens collected 1 week after the start of treatment (4 treatments) were subjected to PD analysis to assess single- and combination-treatment efficacy and to generate guidelines for the design of a second study in an orthotopic primary patient-derived xenograft (PDX) model. YKL-5-124 as a single agent and in combination with JQ1, demonstrated near-complete CDK7 engagement in spleen, liver as well as tumor tissues following one-week of treatment (**Supplementary Figure 5C**). Immunohistological examination of xenograft tumors demonstrated that YKL-5-124 and JQ1 each led to inhibition of cell proliferation, increased apoptosis and downregulation of MYCN, with much more striking effects seen with the combination (**Figure 5C**).

We next tested whether the combination would show similar synergy in the SFNB17 PDX model. In this case, we switched to three times weekly (Monday, Wednesday, Friday) dosing, leaving a weekend “holiday” which avoided the weight loss observed in the established cell line xenograft study. Mice were treated for a total of 28 days (14 doses) or until the tumor size exceeded 2000 mm^3^. Similar to our results in the cell line xenograft model, while JQ1 and YKL-5-124 led to minimal to moderate effects respectively, the combination completely suppressed tumor growth (**Figure 5D**). In this model, only the combination-treated cohort exhibited significantly increased survival (p=0.004) (**Figure 5E**). Tumors analyzed after two weeks (6 treatments) of treatment showed full CDK7 engagement in YKL-5-124 and combination-treated animals (**Figure 5F**), with similar effects as the established cell line tumors on IHC analysis (**Supplementary Figure 5D**) Altogether, compared with each single agent, combined treatment resulted in a more pronounced therapeutic effect in mouse models of NB.

## Discussion

We demonstrate that CDK7 inhibition with the novel, highly selective inhibitor, YKL-5-124 results in aberrant cell cycle progression and without the induction of apoptosis in NB cells. Consistent with previous reports in other models (18, 26), YKL-5-124 in NB cells inhibited CDK1 and CDK2 activation, but had minimal effects on Pol II-mediated transcription. Interestingly, YKL-5-124 induced G1-S progression defects in *MYCN*-amplified but not in *MYCN* nonamplified NB cells. This discrepancy could be explained by the heterogeneity in cell cycle checkpoint activation that has been reported in NB cells (31). We show that YKL-5-124 strongly synergizes with inhibitors of BRD4 *in vitro* and in *in vivo* preclinical models of NB. The combination affected the expression of genes associated with transcription, including the *MYCN* signature and ribosome biogenesis, and likely underlies the observed synergistic cell death. Moreover, the transcriptional changes following BRD4 inhibition identified an upregulated gene signature enriched for genes involved in G2/M cell cycle transition that may be associated with the development of resistance if used as a single agent in clinical trials, but which leads to a dependency on CDK7 function.

Previous studies have reported that inhibition of CDK7 with THZ1 affected the expression of essential transcription factors (TFs) that form the transcriptional core regulatory circuitry (CRC) in NB and that the addition of the BRD4 inhibitor, JQ1 further potentiated this effect (32). We observe that selective inhibition of CDK7 with YKL-5-124 did not affect the expression of these TFs in NB cells or global transcription for that matter, suggesting that the effects observed with THZ1 could be due to its combined targeting of CDKs 7/12/13 and pointing to compensation by other transcription-associated CDKs. This conclusion is supported by the observation that the combination of CDK7 and CDK12 or CDK9 inhibitors reduced Pol II phosphorylation and MYCN levels (this study) (4, 33). In addition, we demonstrated that YKL-5-124 had only a minor effect on an established *MYCN* gene signature (30) and that inhibition of other components of the transcription machinery such as BRD4 is required to suppress MYCN-driven transcriptional program. Whether the observed effect could be reproduced in *MYCN*-nonamplified but MYC overexpressing NB cells and thus explain the synergistic effect of CDK7 and BRD4 inhibition remains to be seen in future studies. The lack of effect on Pol II CTD phosphorylation with YKL-5-124 contrasts sharply with recent findings that CDK7, when functioning as part of TFIIH was unable to efficiently phosphorylate any substrate tested except for Pol II, while it efficiently activated other substrates (including CDKs 9/12/13) as well as the Pol II CTD, as part of the CAK complex (34). Reduced expression of these TFs was seen also following the addition of JQ1, suggesting that inhibition of transcription initiation and elongation together may be required to decrease the expression of essential TFs and, consequently, global Pol II transcription. Thus, CDK7 inhibition appears to be necessary but not sufficient to disrupt the survival programs that are active in NB cells, likely due to functional compensation by other transcription-associated kinases. Alternatively, we cannot exclude that CDK7 could regulate transcription at another level, for example, by transcription factor phosphorylation.

JQ1 was previously reported to cause MYCN-dependent growth inhibition and apoptosis in NB models (8). In our hands, JQ1 showed modest anti-proliferative activity with sub-micromolar to above 10 micromolar IC_50_ values in a cell-type dependent manner. A recent study reported that combined CDK7/12/13 inhibition with THZ1 and BRD4 inhibition overcomes enhancer reprogramming in BET inhibitor-resistant leukemia cells and consequently suppresses oncogenic transcription (35). Whether selective CDK7 inhibition with YKL-5-124 combined with BRD4 inhibition synergistically targets enhancer plasticity in NB cells requires further investigation.

Our results provide preclinical evidence for targeting CDK7 in combination with epigenetic therapies relying on BRD4 inhibition in the treatment of patients with NB. Additionally, this strategy may prevent the emergence of resistance to BRD4 inhibitors, which are currently being tested in Phase 1 trials in pediatric patients with solid tumors, including neuroblastoma.

## Data Availability Statement

RNA-seq datasets have been deposited in the Gene Expression Omnibus (GEO), accession number GSE183587. All other data are available from the corresponding author upon request.

## Ethics Statement

The animal study was reviewed and approved by the Institutional Animal Care Use Committee (IACUC) of the University of California, San Francisco (UCSF).

## Author Contributions

Conception and design: YG, NG, CG, and RG. Development of methodology: YG, MK, NN, and SD. Acquisition of data: YG, MK, and NN. Analysis and interpretation of data: YG, MK, NN, SD, and BA. Writing, review, and/or revision of the manuscript: YG, MK, MV, NN, SD, BA, NK, TZ, NG, CG, and RG. Administrative, technical, or material support: YG, MK, NN, MV, NK, TZ, NG, and CG. Study supervision: NG, CG, and RG.

## Funding

This work was supported by a Friends for Life Neuroblastoma Fellowship (YG), the Rally Foundation for Childhood Cancer Research (MK), a DOD TTSA CA150634 (RG, CG, NG) and NIH R01 CA197336 (RG).

## Conflict of Interest

NG is a founder, science advisory board member (SAB), and equity holder in Syros, C4, Allorion, Jengu, B2S, Inception, EoCys, CobroVentures, GSK, Larkspur (board member), and Soltego (board member). The Gray lab receives or has received research funding from Novartis, Takeda, Astellas, Taiho, Jansen, Kinogen, Arbella, Deerfield, Springworks, Interline and Sanofi. TZ is a consultant and equity holder and founder of EoCys. BA is a shareholder in Syros Pharmaceuticals.

The remaining authors declare that the research was conducted in the absence of any commercial or financial relationships that could be construed as a potential conflict of interest.

The reviewer, MI, declared a past co-authorship with one of the authors, RG, to the handling editor.

## Publisher’s Note

All claims expressed in this article are solely those of the authors and do not necessarily represent those of their affiliated organizations, or those of the publisher, the editors and the reviewers. Any product that may be evaluated in this article, or claim that may be made by its manufacturer, is not guaranteed or endorsed by the publisher.
