# Supplementary material for: Synergistic Anti-Tumor Effect of Combining Selective CDK7 and BRD4 Inhibition in Neuroblastoma
Source: Front Oncol. 2022 Jan 27;11:773186. doi: 10.3389/fonc.2021.773186 (PMC8859926; doi:10.3389/fonc.2021.773186)
Supplement: Supplementary file 1 [file DataSheet_1.pdf]

Supplementary Figures

A

IC$_{50}$ (nM) values	YKL-5-124	YKL-5-124R
Kelly	24.5	1544
IMR-32	22.5	>5000
IMR-5	8.4	1605
SK-N-BE2	25	
LAN-5	23	
SK-N-DZ	60	
SH-SY5Y	6.86	892
CHLA-20	15.0	1714
SK-N-AS	8.5	842

B

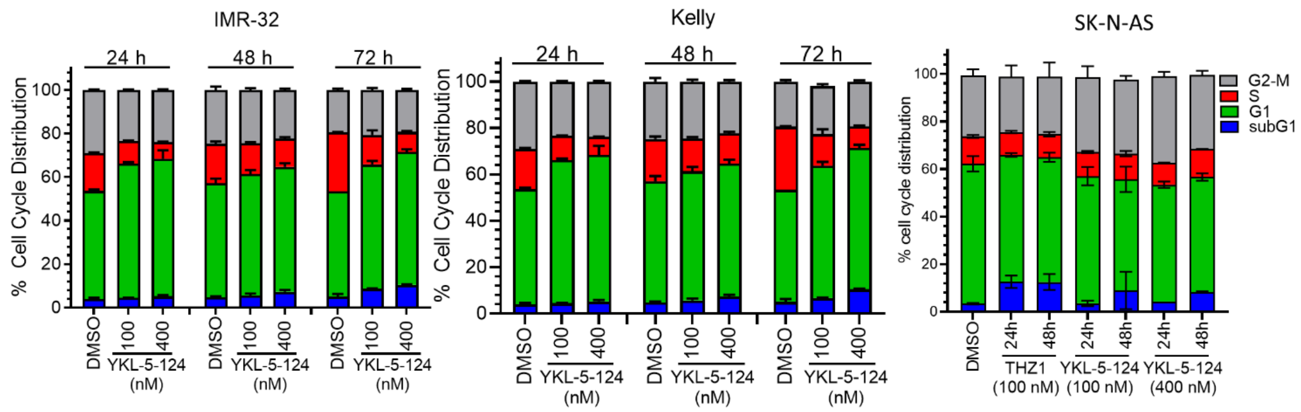

C

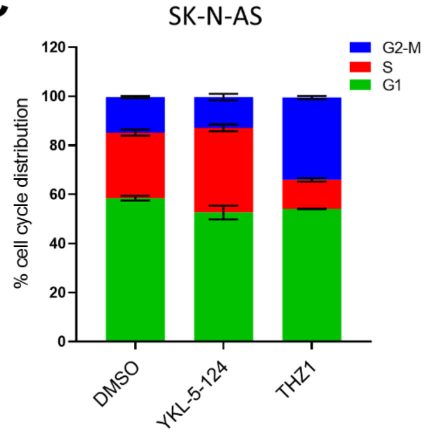

Supplementary Figure 1. Inhibition of CDK7 with YKL-5-124 is cytostatic in NB cells. (A) IC$_{50}$ values of YKL-5-124 and YKL-5-124R in NB cells at 72 h**. (B)** Cell-cycle analysis of *MYCN*-amplified (Kelly, IMR-32) and *MYCN*-nonamplified (SK-N-AS) NB cells exposed to YKL-5-124, THZ1 at the indicated concentrations and time points by flow cytometry with propidium iodide (PI) staining. **(C)** Flow cytometry analysis of EdU staining in SK-N-AS cells treated with 100 nM THZ1, 100 nM YKL-5-124, or DMSO for 24 h. Quantification of staining, data represents mean ± SD; $n = 3$.

Mechanism	ID	Ref.	Targets	IC_{50} (nM)			Synergy with YKL-5-124
				IMR-32	Kelly	LAN-5	
DNA methylation	RG-108	1	DNMT	>10000	>10000	>10000	NO
Histone acetylation	C646	4	p300/CREB-binding protein (CBP)	4093	>10000	>10000	NO
	UNC0638	2	G9A (EHMT2) and GLP1 (EHMT1) (on H3K9)	3711	~2500	2060	NO
	GSK-J4	10	JMJD3 (on H3K27)	3484	4096	2759	NO
	GSK343	3	EZH2	>10000	8913	>10000	NO
Histone deacetylation	SAHA	5	HDAC (10 nM)	619	772	1637	Additive
DNA damage, chromatin remodeling	Iniparib	7	PARP-1	>10000	>10000	>10000	NO
Chromatin reading	JQ1	12	Brd4 (77 nM for BET domain)	1058	>10000	314.7	Strong synergy*
	iBET726	8	Brd2/3/4 (35 nM)	982	815	619	Strong synergy*
	iBET151	9	Brd2/3/4 (0.5/0.25/0.79 uM)	711	692	790	Strong synergy*

*Strong synergy: CI values are < 0.3

Supplementary Figure 2. YKL-5-124 is synergistic with inhibitors of BRD4. Summary of IC_{50} values of inhibitors of epigenetic and chromatin modulators and their synergistic effects with YKL-5-124 in NB cells.

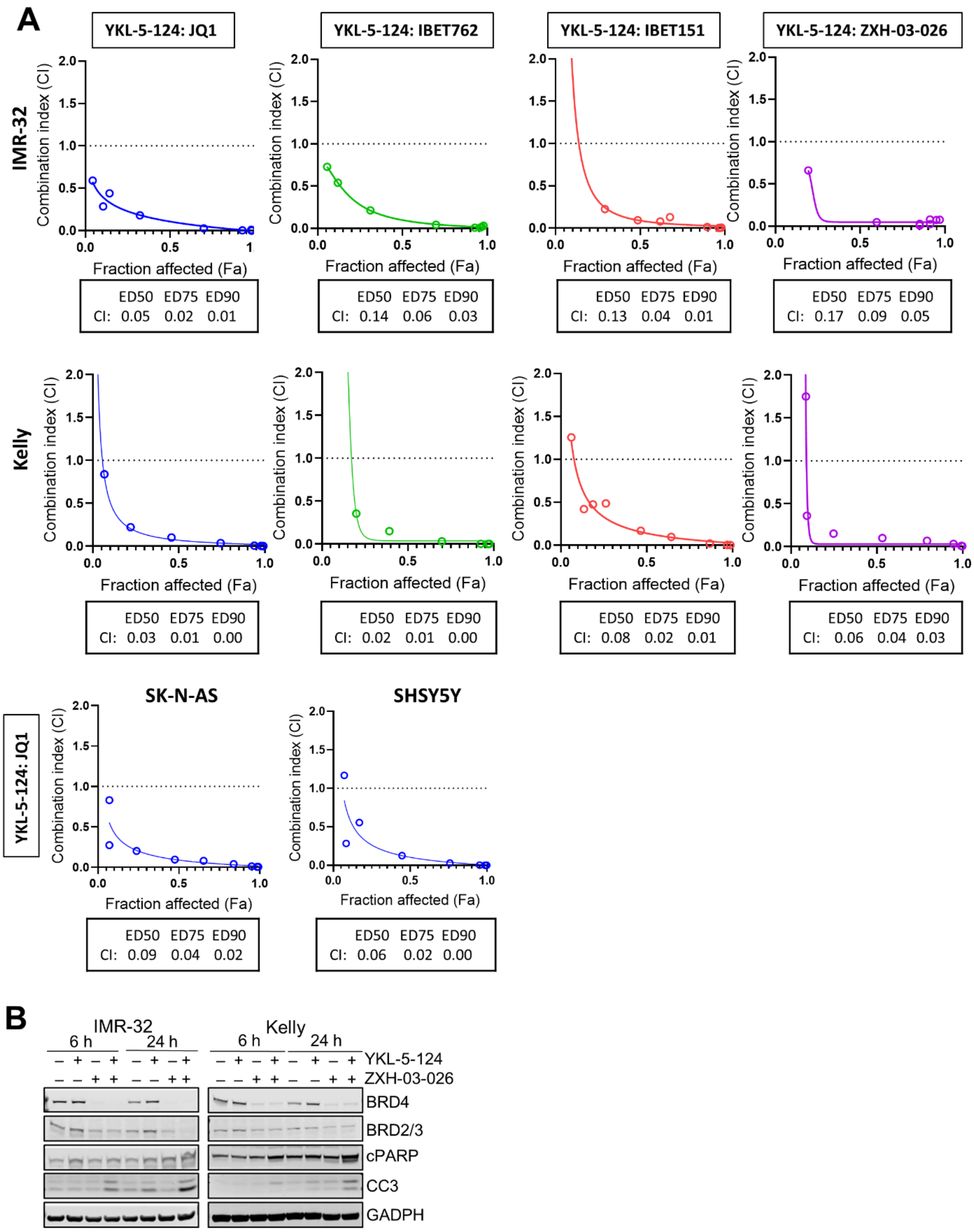

Supplementary Figure 3. Synergistic effect of dual CDK7 and BRD4 inhibition. **(A)** Combination-Index analysis. Fraction affected (Fa) versus combination index (CI) plots were generated using the method of Chou and Talalay (22) to determine the extent of synergy for a combination of YKL-5-124 and indicated BRD4 inhibitor in *MYCN*-amplified (Kelly, IMR-32), *MYCN*-nonamplified (SK-N-AS) NB cells and BJ and IMR-90 fibroblast cells treated as described in Fig. 3A. CI < 1 indicates a synergistic effect, CI > 1 an antagonistic effect, and CI = 1 an additive effect. The line in each plot indicates a reference point of a CI value of 1. **(B)** WB analysis of apoptosis marker in NB cells treated with 100 nM YKL-5-124 and 400 nM ZXH-03-026 or the combination at the indicated time points.

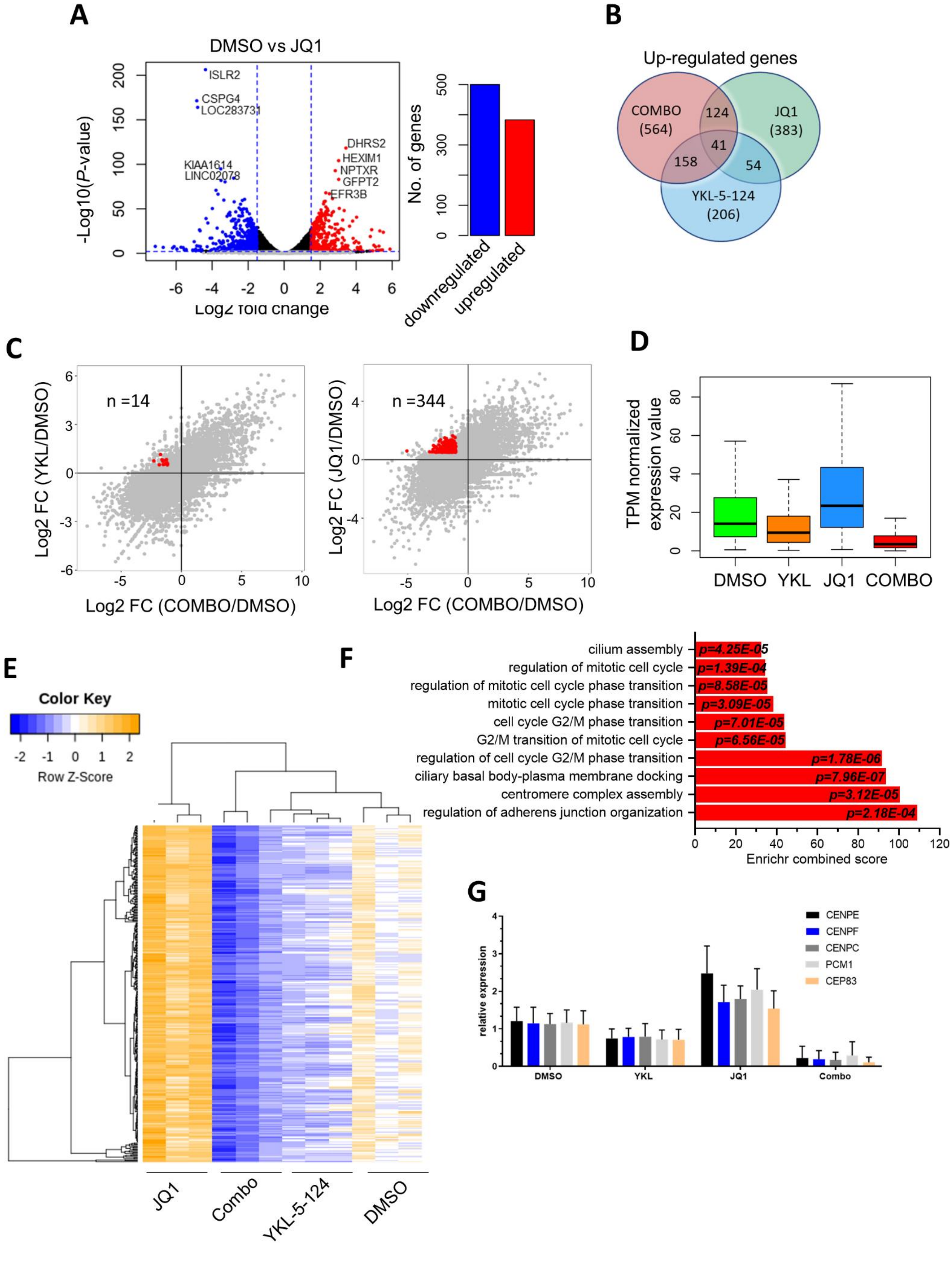
A
DMSO vs JQ1
ISLR2
CSPG4
LOC283731
KIAA1614
LINC02078
DHRS2
HEXIM1
NPTXR
GFPT2
EFR3B
-Log10(P-value)
Log2 fold change
No. of genes
downregulated
upregulated
B
Up-regulated genes
COMBO (564)
JQ1 (383)
YKL-5-124 (206)
124
41
158
54
C
n =14
n =344
Log2 FC (YKL/DMSO)
Log2 FC (JQ1/DMSO)
Log2 FC (COMBO/DMSO)
D
TPM normalized expression value
DMSO
YKL
JQ1
COMBO
E
Color Key
Row Z-Score
JQ1
Combo
YKL-5-124
DMSO
F
cilium assembly p=4.25E-05
regulation of mitotic cell cycle p=1.39E-04
regulation of mitotic cell cycle phase transition p=8.58E-05
mitotic cell cycle phase transition p=3.09E-05
cell cycle G2/M phase transition p=7.01E-05
G2/M transition of mitotic cell cycle p=6.56E-05
regulation of cell cycle G2/M phase transition p=1.78E-06
ciliary basal body-plasma membrane docking p=7.96E-07
centromere complex assembly p=3.12E-05
regulation of adherens junction organization p=2.18E-04
Enrichr combined score
G
CENPE
CENPF
CENPC
PCM1
CEP83
relative expression
DMSO
YKL
JQ1
Combo

Supplementary Figure 4. Combined CDK7 and BRD4 inhibition leads to downstream effects on transcription. **(A)** Volcano plot representation of differentially expressed genes following treatment with 400 nM JQ1 for 4 h (*left*). The fold changes are represented in log2 scale (x-axis) and the −log10 *P*-value depicted on the y-axis (FDR < 0.01 and log2 FC >1.5). Bar plot depicting the numbers of up- and downregulated genes (*right*). **(B)** Venn diagram showing upregulated genes (FC >1.5 and FDR ≤ 0.01) in NB cells treated with 100 nM YKL-5-124, 400 nM JQ1 or the combination. Rows represent z-scores of $\log_2$ expression values for each gene. **(C)** Scatter plot showing the correlation between fold changes in gene expression in NB cells treated with YKL-5-124 *(left)* or JQ1 *(right)* as single agents or the combination. Genes significantly downregulated with combination treatment and upregulated with YKL-5-124 or JQ1 are highlighted in red. **(D)** Box plot depicting the change in expression of 344 transcripts that were upregulated following single-agent JQ1 treatment and were downregulated with the combination (FDR < 0.01). **(E)** Heat map of gene expression values of 344 genes upregulated following treatment with JQ1 and downregulated following the combination. **(F)** GO enrichment analysis of the genes shown in **E. (G)** RT-qPCR analysis of selected genes identified in F, in cells treated as in B.

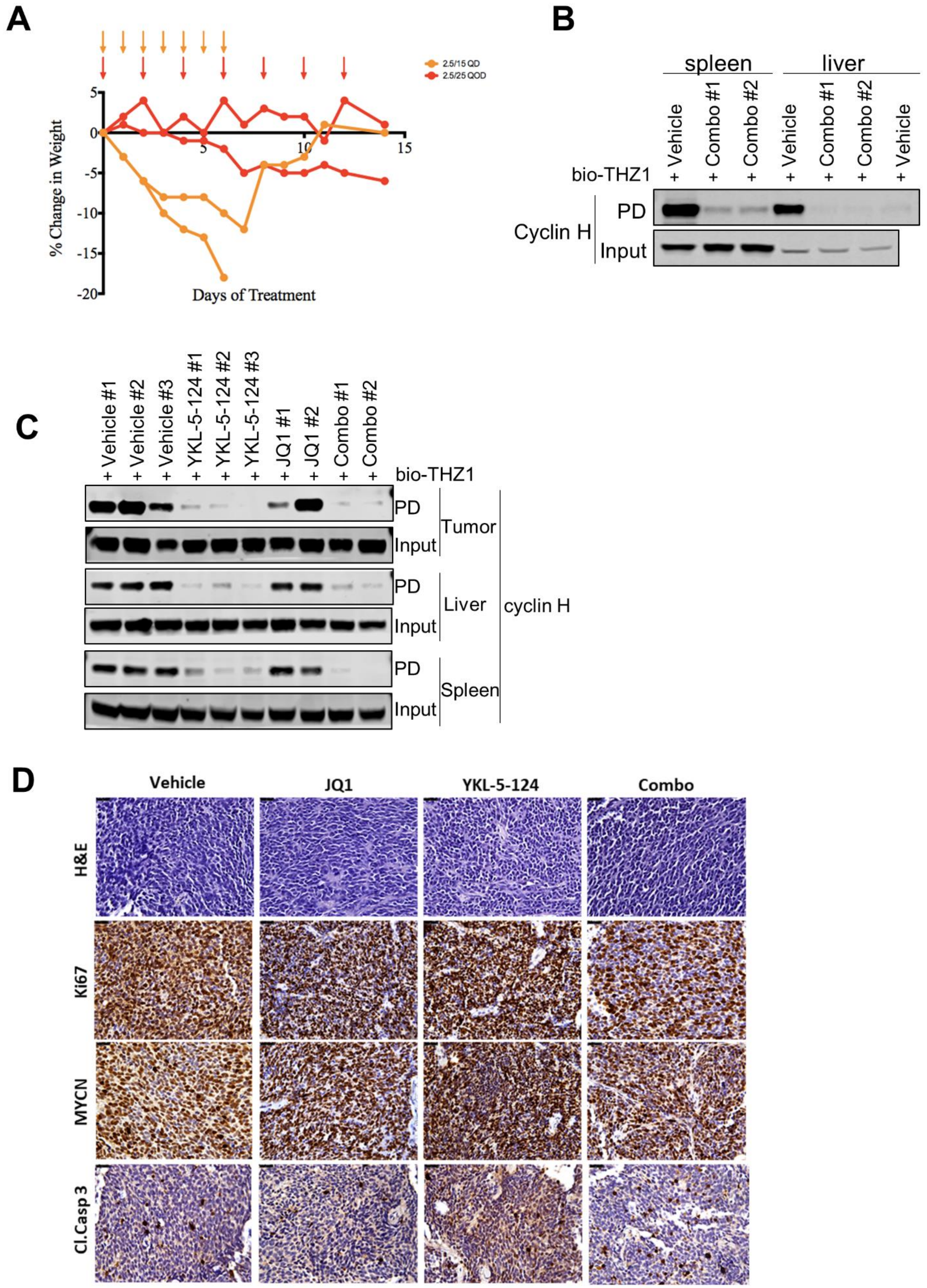

Supplementary Figure 5. Synergistic anti-tumor effect of YKL-5-124 and JQ1. (A) Body weights of mice treated with 2.5 mg/kg YKL-5-124, 25 mg/kg JQ1, and YKL-5-124 or vehicle control (5DW +10% DMSO +

10% HP-beta-cyclodextrin) three times per week for 24 days. Mean ± SD values are presented. **(B)** Analysis of target engagement in liver and spleen tissues of non-tumor-bearing mice treated with the combination of YKL-5-124 and JQ1 as described in **A**. Lysates prepared from homogenized tissue were incubated with bio-THZ1 for 24 h at 4 °C and immunoprecipitated proteins were analyzed for cyclin H via WB. **(C)** Analysis of target engagement in tumor, liver, and spleen tissues from mice bearing subcutaneous human NB cell line xenografts and treated with YKL-5-124 or JQ1 or the combination. Lysates prepared from homogenized tissue were incubated with bio-THZ1 as in B and immunoprecipitated proteins analyzed for cyclin H via WB. **(D)** IHC analysis of morphology (hematoxylin & eosin, H&E), proliferation (Ki67), apoptosis (cleaved caspase 3) and MYCN in PDX tumors harvested from animals treated as described in A for 7 days. (Magnification, 40X).
